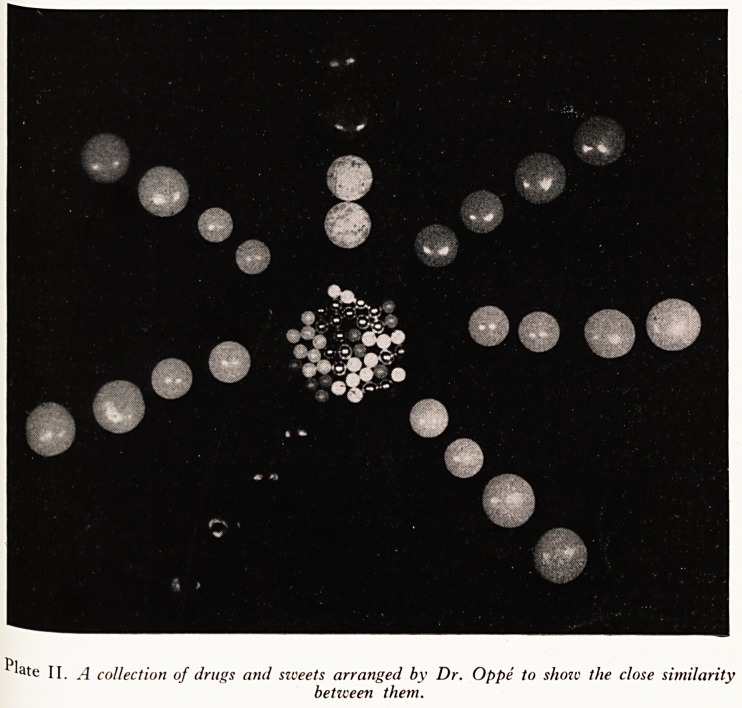# Accidental Poisoning with the Antihistamine Drug "Histantin"

**Published:** 1958-04

**Authors:** T. F. Hewer


### ACCIDENTAL POISONING WITH THE ANTIHISTAMINE DRUG "HISTANTIN"

*A Clinical Pathological Conference of the University of Bristol Medical School.*

CHAIRMAN: PROFESSOR T. F. HEWER

*Professor Hewer.* I will ask Dr. Apley, who looked after this child, to give us the clinical history.

*Dr. John Apley.* Let me first tell you about the family of this little girl, an only child, who died in hospital three days before her first birthday. The father is a man in his early thirties, who owns a shop. The mother is a kind and pleasant woman of some twenty years of age. They live in a small house over the shop. Mother has a mild skin disorder for which, unfortunately, as events will show, she was taking anti-histamine tablets ("Histantin").

She came into the room where Jane had been left playing one day and found her eating some tablets. Mother thinks that nine were swallowed in all. The child was seen by a doctor about one hour later. There had been no vomiting and Jane seemed well, so the doctor advised that she be taken home to rest. Fifteen minutes later she started to vomit and had generalised convulsions.

She was immediately taken to the local hospital where her stomach was washed out with bicarbonate of soda solution, approximately one-and-three-quarters hour after ingestion of the tablets. While this was proceeding the doctor telephoned me. With a recent previous experience of such a case (a child who also died despite treatment) I advised that Jane should be brought into hospital immediately. The journey was a long one and she arrived in hospital some four hours after swallowing the tablets.

On admission she was slightly cyanosed and generalised muscular twitching was seen. Her temperature was 100·8°. The heart rate varied between 80 and 170 and was irregular (presumably owing to the so-called quinidine effect of the drug). Moist sounds were heard all over the lungs, an ominous sign which carries a very grave prognosis in this condition.

### TREATMENT AND PROGRESS

The stomach was sucked out, and some paraldehyde was injected intramuscularly. She was put in an oxygen tent. We were faced here with the problem of dealing with a condition in which central nervous depression may prove lethal, yet nervous stimulation may produce fits which are themselves highly dangerous. The problem of stimulants versus anticonvulsants is an insoluble one, but in this case, as in other cases of poisoning, the usual rule is to treat the immediate disturbance rather than the underlying condition. Some forty-five minutes later, when the generalised twitching was still marked, she was given rectal thiopentone.

Three hours after admission the heart rate was rapid and irregular, she was vomiting thick brown material, and the twitching persisted. After a further small rectal injection of thiopentone the twitching ceased, her colour became better and the respiratory rate was lower.

At this stage, the case was discussed with Professor Heller, who kindly came to the hospital to see the child. Seven hours after admission convulsions recurred and further mild sedation was attempted. In spite of treatment the twitching persisted, her temperature rose to 105°, her general condition deteriorated and she developed peripheral circulatory failure and died some thirteen hours after admission to hospital.

Professor Hewer, shall I say a few words now or later about the natural history of poisoning in children?

*Professor Hewer.* I think we should like to hear it now.

*Dr. Apley.* In paediatrics, as in general medicine, we tend to congratulate ourselves on the fact that the incidence and dangers of many disorders, especially infections, are being rapidly controlled. Poisoning and accidents, in the home or on the road, are unfortunately not decreasing. I may add that dangerous iatrogenic disorders also seem to be increasing.

Medicines or pills are the cause of about half the deaths in children due to drugs. Only very rarely is this due to an error or accident by the doctor or chemist. The others are due to the child swallowing liniments, cleaners, polishes, pesticides, etc.

The most dangerous room in the house is the kitchen, followed in order by the bedroom, bathroom, and living room. The type of children affected can be divided into two groups. The first, and perhaps the most tragic, is the group of normal toddlers with initiative and an urge to explore. In children, of course, the tongue is an organ of sense. Any moveable object may be put into the mouth, but is often spat out again unless it is tasty. The difficulty is that more and more drugs are being made attractive to look at and palatable to swallow. Some of the NHS prescriptions are flavoured with extract of cherries, and so on, and some tablets are so like "Smarties" and "Hundreds and Thousands", used to decorate cakes, that any child might be expected to gobble them up (here a coloured slide, lent by Dr. Oppé, was shown to illustrate the similarity between tablets and sweets, (Plate II)). The second group, possibly larger than the first, is made up of infants and toddlers who eat anything. They are fierce feeders, both off the bottle or the breast, and will chew thumbs, fingers or anything they can get hold of. This is said to be a form of self-gratification and has been claimed to be part of a widespread psychological disturbance.

*Professor Hewer.* Before we have any discussion of this case I shall ask Dr. Halford to tell us his autopsy findings.

*Dr. M. E. H. Halford.* The autopsy does not give us very much additional information. There was some dehydration, with inelasticity of the skin. The liver, although normal in size, was flabby and pale. Histological examination confirmed that there was diffuse fatty change and also some early centrilobular necrosis. The kidneys showed advanced cloudy swelling, but this may have been a post-mortem change since the autopsy was performed twenty-three hours after death. The brain was moderately oedematous, but there were no petechial haemorrhages.

There was no evidence of pre-existing disease or congenital deformity.

The Registrar-General's returns for England and Wales show the changing incidence of fatal poisoning in children under ten years of age, emphasising Dr. Apley's comments. During the period 1931–1935 the commonest causes were:

| | | | |
|---|---|---|---|
| Belladonna .. .. | 8 cases | Disinfectants .. .. | 5 cases |
| Caustic soda .. | 7 ,, | Camphor .. .. | 5 ,, |
| Strychnine .. .. | 6 ,, | Aspirin .. .. | 3 ,, |

From 1950–1952 the list is very different:

| | | | |
|---|---|---|---|
| Ferrous sulphate .. | 24 cases | Strychnine .. .. | 12 cases |
| Antihistamines .. | 19 ,, | Lead .. .. .. | 7 ,, |
| Aspirin .. .. | 15 ,, | Oil of Wintergreen .. | 6 ,, |

When allowance is made for the shorter second period, the increase is considerable, and antihistamine drugs are second on the list.

There are two points of interest in the course of the illness of this child. One which emerged during the inquest was that the first abnormality noticed was a vacant expression. This has been observed in other reported cases of antihistamine poisoning. The second point is the occurrence of vomiting; this is unusual. In the one reported case of Histantin poisoning[3] vomiting also occurred. Perhaps Professor Heller will be able to explain these points. The reported case was successfully treated by light sedation atropine and amphetamine, although a large amount of the drug (900 mgm.) had been ingested.

*Dr. Apley.* I don't believe Dr. Halford told us anything about the lungs.

PLATE II

Plate II. *A collection of drugs and sweets arranged by Dr. Oppé to show the close similarity between them.*

*Dr. Halford.* The trachea and bronchi contained a small quantity of slightly bloodstained watery fluid. There was no pulmonary oedema. The histology of the lung showed the bronchiolar epithelial cells to be distended with mucin. This increased secretion, in the absence of pulmonary oedema could account for the accumulation of fluid in the upper respiratory tract. This excess of fluid has been reported in other cases of antihistamine poisoning.

*Professor Hewer.* If the baby's mother had not found the bottle of Histantin, and realised that the baby had swallowed some, the clinicians and the pathologist would have had great difficulty in determining the cause of the illness. I should like to put it to Dr. Halford that he would have had no clue.

*Dr. Halford.* It would be very difficult indeed to diagnose. The vacant stare, lack of vomiting, muscle twitching with some convulsions and increased upper respiratory tract secretions might suggest antihistamine poisoning if one happened to think of it. I could not have proved it at autopsy without chemical analysis.

*Dr. Apley.* Professor Hewer's remark raises a very important issue. In this case it was obvious that the child had swallowed something poisonous, but if the evidence has been destroyed the diagnosis must often be completely unsuspected. I saw a most tragic example two or three years ago, when I was visiting a large hospital, a long way from here, to see some cases. While I was discussing them with the local paediatrician an infant was brought in, vomiting blood and moribund. He died shortly afterwards. I was told that only one month previously his elder brother had died, also after vomiting blood. The possibility of poisoning had not been confirmed; on post-mortem examination acute haemorrhagic gastritis was found, and it was suggested that this had been due to a streptococcal infection.

We took the father into a quiet room and cross-examined him. Eventually it came out that the mother was pregnant again and was taking ferrous sulphate tablets. The local doctor was telephoned, hurried round to the house, and found that a large number of ferrous sulphate tablets were missing. Ferrous sulphate poisoning, previously unsuspected, had killed both the children.

*Professor H. Heller:* Did you find any reference in the literature, Dr. Halford, to lesions in the liver?

*Dr. Halford.* In the reported cases, post-mortem findings are indifferently recorded, and sometimes not mentioned. In others gross changes are described and very few give a full histological description. The liver is mentioned as being congested in one case but this was part of a generalised venous congestion. Findings in other cases include cerebral oedema with petechial haemorrhages, petechiae in the thymus and pericardium, and, as I have already mentioned, severe upper respiratory tract catarrh.

*Dr. A. C. Hunt:* I would like to make a comment on why the Coroner did not have an analysis made after this post mortem. In this case he is quite justified from his point of view in not needing a chemical analysis, as the facts are quite clear. In some cases, for example when the amount of a drug taken is not definitely known, and if there is any natural disease that might have influenced death, it is most important that the pathologist should insist on an analysis, made either by himself or by the Forensic Science Laboratory.

*Professor Hewer.* Will Professor Heller tell us about the toxic effects of the antihistamines?

*Professor Heller:* I am afraid there is comparatively little I can contribute to this interesting discussion. However, two remarks may be worth making: First, the absence of vomiting in most patients suffering from poisoning with antihistamines is very likely due to a depressant effect of the drug on the so-called chemoreceptor trigger zone in the floor of the fourth ventricle. You will remember that some of the antihistamine compounds as well as chlorpromazine (to which they are chemically closely related) are actually used for their anti-emetic properties. There is, on the other hand, some indication that antihistamine may have a direct irritant effect on the gastric mucosa. For instance, Drs. Ashford, Smart and I[1], in an investigation concerned with the effect of antihistamines on gastric secretion, found that ulcer patients did tolerate these drugs rather badly and responded occasionally with an increase in gastric secretion. One may therefore conjecture that vomiting in a poisoned patient depends on the "competition" between central and peripheral action of the antihistamine drug. Secondly, the action of the antihistamine on the eyes has been mentioned. One would expect such effects since the antihistamines have quite pronounced atropine- (or perhaps more correctly hyoscine) like properties.

This brings me to central effects of the antihistamines. They, as Dr. Apley has already stressed, seem to consist in a curious mixture of depression and stimulation. It has been suggested that the stimulant action is related to the atropine-like side effect and it is of course true that belladonna poisoning may lead to central excitation and pyrexia. But it should not be forgotten that antihistamines are also quite strong local anaesthetics and that local anaesthetics like cocaine and procaine have also a pronounced stimulant effect on the central nervous system. However, remembering the patient under discussion whom—thanks to the kindness of Dr. Apley—I was able to see in the Children's Hospital, and the descriptions of some published cases of poisoning with antihistamines, I have the impression that the central effects of these toxic doses consist in a curious mixture of depression and stimulation of different parts of the brain. One is reminded of morphine although the cerebral localisation of these effects is obviously different. The therapeutic problem is therefore—as already mentioned—very difficult. One really does not know which drugs to use to combat the toxic effects on the central nervous system. The history of this patient which, alas, resembles only too closely that of many other cases of antihistamine poisoning in children, has led me to the conclusion that more experimental work is needed to investigate the effects of toxic doses of antihistamines. I have, in fact, last week proposed this subject to a Ph.D. candidate. I think that we shall only arrive at a rational therapy if we know which parts of the central nervous system are differentially affected by toxic doses of antihistamines. Judging again from the comparison with morphine, we need something like nalorphine that is to say a competitive inhibitor.

Lastly, a word about a further feature which was rather prominent in this patient, namely the effects on the heart. The quinidine-like action of antihistamines was first discovered in experiments on the isolated rabbit auricle. The present case suggests that it may not only be a pharmacological curiosity but a serious toxicological feature which requires therapeutical efforts.

*Question*. Is it logical to use histamine as a neurotransmitter for treatment of the cerebral depression?

*Professor Heller*. There is good evidence that histamine is present in certain nerves but little evidence that it occurs in the central nervous system. Thus, it is unlikely to have a function as a central humoral transmitter. It is therefore difficult to see any beneficial effects which injections of histamine could have in antihistamine poisoning so far as the central nervous system is concerned. It may be useful, however, to find out whether any of the peripheral effects of the antihistamines could be antagonized by histamine injections.

*Dr. Apley*. Some say that histamine is useless and others that it should on no account be given.

*Dr. W. O. Spence*. What would be the dosage of antihistamine which should alert the general practitioner to take immediate action? I had a case two weeks ago of a toddler who swallowed about one ounce of Elixir Benadryl (about four times the correct dose); I reassured the mother and left her to report again if any signs appeared, but it would appear as though it would have been safer to have made the child vomit.

*Professor Heller*. It is difficult to lay down maximum doses for the various compounds with antihistaminic effects, not only because these substances have a different potency[2] but also because the sensitivity of different patients to the same antihistamine drug varies widely. It may even vary considerably in the same patient according to his state of health. May I refer you to the work of Warin[4] who has shown by applying intradermal wheal tests that a patient may not be affected by the "ordinary" dose of an antihistaminic, but may need a very much larger dose to affect his allergic condition. However, I should like to stress that the wheal test in such a case is essential.

*Dr. B. E. McConnell.* It is recorded that this child swallowed the drug at about 2 p.m. This was presumably after a meal. Would not an injection of apomorphine have produced satisfactory emesis?

*Professor Heller.* I would assume that apomorphine would be of value at an early stage, e.g., before central depression has occurred. But I think that apomorphine would be dangerous at a later stage since it has a medullary depressant action itself.

*Dr. T. E. Oppé.* Gastric lavage is much more sure and also safer because with the use of an emetic there is a danger of aspiration pneumonia.

*Professor Heller.* I quite agree that an emetic may be dangerous. In that connection I think the simplest way of producing vomiting in a child, namely by insertion of a finger down its throat, should not be forgotten.

*Dr. A. J. Webb.* Emetics are very unpopular in the Casualty Department and gastric lavage is always performed.

*Dr. Halford.* Mention has been made of the minimum lethal dose of antihistamines. In an account of a published series of fatal cases I found the smallest fatal dose was 100 mgm. of methapyrilene hydrochloride ("Thenylene") for a child of sixteen months: this is just twelve times the normal dose for such a child and only twice the adult dose.

### REFERENCES

1 Ashford, C. A., Smart, G. A., and Heller, H., 1949, *Brit. J. Pharmacol.* **4**: 157.

2 Bain, W. A., 1949, *Proc. Roy. Soc. Med.* **42**: 615.

3 Gill-Carey, M. C., 1954, *B.J.M.*, **i**: 688.

4 Warin, R. P., 1950, *Brit. J. Derm.*, **62**: 159.

## Figures and Tables

**Plate II. f1:**